# Evaluation of Radiation Exposure and Influential Factors in Cone-Beam Computed Tomography (CBCT) of the Head and Abdomen during Interventional Procedures

**DOI:** 10.3390/tomography10030025

**Published:** 2024-02-26

**Authors:** Mingming Li, Weiwei Qu, Dong Zhang, Binyan Zhong, Zhi Li, Zhengyu Jiang, Guanyin Ni, Caifang Ni

**Affiliations:** 1 Department of Interventional Radiology, The First Affiliated Hospital of Soochow University, No. 188 Shizi Road, Suzhou 215006, China; mmli@suda.edu.cn (M.L.); zd_sz@163.com (D.Z.); byzhongir@sina.com (B.Z.); lizhisoochow@suda.edu.cn (Z.L.); 20224232033@stu.suda.edu.cn (Z.J.); 20225232247@stu.suda.edu.cn (G.N.); 2 State Key Laboratory of Radiation Medicine and Protection, School for Radiological and Interdisciplinary Sciences (RAD-X) and Collaborative Innovation Center of Radiation Medicine of Jiangsu Higher Education Institutions, Soochow University, Suzhou 215123, China; wwqu@suda.edu.cn

**Keywords:** cone-beam computed tomography, radiation dose, body mass index, gender, field of view, automatic exposure control

## Abstract

Cone-beam computed tomography (CBCT) is a widely used imaging technique in interventional radiology. Although CBCT offers great advantages in terms of improving comprehension of complex angioarchitectures and guiding therapeutic decisions, its additional degree of radiation exposure has also aroused considerable concern. In this study, we aimed to assess radiation exposure and its influential factors in patients undergoing CBCT scans of the head and abdomen during interventional procedures. A total of 752 patients were included in this retrospective study. Dose area product (DAP) and reference air kerma (RAK) were used as measures of patient dose. The results showed that the median values of DAP were 53.8 (50.5–64.4) Gy⋅cm^2^ for head CBCT and 47.4 (39.6–54.3) Gy⋅cm^2^ for that of the abdomen. Male gender and body mass index (BMI) were characterized by increased DAP and RAK values in both head and abdominal CBCT scans. Larger FOV size was associated with a higher DAP but a lower RAK value, especially in head CBCT scans. Exposure parameters under automatic exposure control (AEC) also varied according to patient BMI and gender. In conclusion, the patients received slightly higher radiation doses from head CBCT scans than from those applied to the abdomen. BMI, gender, and FOV size were the key factors that influenced the radiation dose administered to the patients during CBCT scans. Our results may help to define and minimize patients’ exposure to radiation.

## 1. Introduction

Cone-beam computed tomography (CBCT) is a valuable three-dimensional (3D) volumetric imaging technique widely utilized in neuroradiology and interventional oncology [1,2]. It offers significant advantages in terms of visualizing complex anatomical structures, guiding percutaneous punctures and catheterizations, verifying intracranial stent placement in real time, and promptly monitoring intracranial hemorrhages or infractions [3,4,5,6,7,8]. However, a notable drawback of this technique is the additional degree of radiation exposure, especially when multiple CBCT scans are performed [9,10,11].

While previous studies have explored the role of CBCT in interventional procedures [3,6,10], there has been limited research specifically focusing on the radiation dose associated with CBCT image acquisition. Some studies have employed phantoms to estimate the dose of CBCT, but the corresponding results have shown significant variations due to the different methodologies used for estimating the effective dose (ED) [12,13,14,15,16,17]. Direct measurement of ED may be affected by differences in phantom size or material. ED measured by Monte Carlo simulations may vary when different parameters and assumptions are used. ED values derived from DAP using a conversion factor (CF) are accurate only when the same phantom size and radiation field conditions are used. Furthermore, using phantoms may not accurately represent the actual patient population, as phantoms may have a relatively low body mass index (BMI) and often lack arms, and, thus, doses derived from phantoms tend to be lower than the clinical doses received by patients [15]. This result underscores the necessity of further investigation into the clinical doses of CBCT. Although patient characteristics, exposure parameters, and field-of-view (FOV) size have been shown to be possible factors that increase the radiation dose from fluoroscopy and 2D angiography [15,16,18,19,20,21], the effects of these factors on the CBCT radiation dose administered to different parts of the body are not well understood due to differences in the modulation of 2D versus 3D imaging parameters by the imaging device. Therefore, there is a need to explore and evaluate the factors affecting CBCT radiation dose and exposure parameters to optimize radiation safety and provide a clinical reference for further research into more dose-efficient imaging protocols.

In this study, we aimed to assess radiation doses and affecting factors with respect to patients undergoing CBCT imaging of the head and abdomen during interventional procedures, analyze the impact of these factors on the exposure parameters, and provide a valuable reference for optimizing radiation doses in interventional procedures.

## 2. Materials and Methods

### 2.1. Study Population

This retrospective study was approved by the institutional review board of the participating hospital. The opportunity to opt out was presented to patients. The requirement for informed consent was waived due to the retrospective nature of this study. Dose data were collected consecutively between January 2022 and June 2023 from patients who underwent abdominal CBCT scans during oncologic interventions and head CBCT scans during neurointerventional procedures. To avoid the effect of metal on radiation dose, patients with metallic implants or cement injections in the irradiated field of view were excluded.

### 2.2. CBCT System and Protocols

All intraprocedural CBCT images were captured using a floor-mounted multiaxis robotic C-arm angiography system (Artis Zeego, VC21C; Siemens Healthcare, Forchheim, Germany). This system was equipped with a 30 × 40 cm flat panel detector (FPD) that allowed C-arm CBCT application via isocentric rotation (Syngo DynaCT, Siemens Healthcare, Forchheim, Germany) and employed an automatic exposure control (AEC) system, which was set by the manufacturer. The AEC adjusted the exposure settings (exposure time, tube current, and tube voltage) with reference to the detector entrance dose (DED) [22]. The user was unable to interact with the AEC but was allowed to set the FOV size in the CBCT scan. The parameters of the imaging protocols were set by a product specialist of Siemens Healthineers Limited (Shanghai, China). Abdominal CBCT was performed utilizing a 6 s dynamic computed tomography (6sDCT-Body) protocol, while for head CBCT, a 20 s dynamic computed tomography (20sDCT-Head) protocol was followed. The FOV size used for abdominal CBCT was 48 cm, while head CBCT was available with either 42 cm or 48 cm FOV sizes, as needed. The acquired projection images were automatically transferred to a dedicated workstation (Syngo X Workplace, Siemens Healthcare, Forchheim, Germany) for image data reconstruction. Table 1 shows the imaging parameters set before the examination.

### 2.3. Patient Radiation Exposure Measurement

Dose area product (DAP) and reference air kerma (RAK) were used as measures of patient radiation dose. DAP is defined as the integral of air kerma over the area of the X-ray beam in a plane perpendicular to the beam axis. RAK, as defined by the International Electrotechnical Commission (IEC), represents the air kerma at the patient entrance reference point (15 cm from the isocenter in the direction of the focal spot). The reference point moves with the gantry. The DAP was measured using an online dosimetric ionization chamber (DIAMENTOR; PTW, Freiburg, Germany), while the RAK was calculated from the DAP measurement. The dose-measuring ionization chamber was calibrated periodically by Siemens Healthineers Limited (Shanghai, China) to ensure measurement accuracy. These dose data were automatically transmitted to the imaging system console, where an X-ray radiation dose structured report (RDSR) was generated upon completion of the imaging exams. Tube voltage (kV) and tube current (mA), as exposure parameters for CBCT, were also recorded in the RDSR. These parameters were modulated via AEC with reference to the detector entrance dose (DED) [22]. While these parameters were set to specific values in the imaging protocols, they could deviate from the ideal range depending on the patient’s body thickness.

### 2.4. Data Collection and Outcomes

The variables analyzed included patient age, gender, BMI, and FOV size. BMI was fitted as both a continuous and categorical variable. According to the BMI for Asian adults proposed by the World Health Organization (WHO) [23], the patients were classified into the following subgroups: <18.5—underweight; 18.5–22.9—normal range; 23.0–24.9—overweight; 25.0–29.9—class I obesity; and ≥30.0—class II obesity. The primary outcome was the DAP value of intraprocedural CBCT and its affecting factors. The secondary outcomes included reference air kerma, tube voltage, tube current, and their influencing factors. Modeling was performed to assess the influence of each independent variable on the DAP values.

### 2.5. Statistical Analysis

Analyses were performed using SPSS statistical software (version 27; SPSS Inc., Chicago, IL, USA). Data were displayed using GraphPad Prism 9 software (GraphPad Software, San Diego, CA, USA). The Shapiro–Wilk test was used to determine the normal distribution of continuous variables. Normally distributed continuous variables were expressed as means ± SD. Non-normally distributed continuous variables were expressed as medians (25th–75th percentile). Categorical variables were expressed as counts (%frequency). Comparisons of radiation dose (DAP and RAK) and parameters (tube voltage and current) between two groups were performed using the independent samples *t*-test (normal distribution of dataset) or the Mann–Whitney *U*-test (non-normal distribution of dataset). When comparisons were performed among the five BMI categories, the ANOVA analysis (for a normal distribution of a dataset) or the Kruskal–Wallis *H*-test (for a non-normal distribution of a dataset) was used. To assess the influence of each independent variable, a multiple regression analysis was conducted, using the DAP value as the response and testing BMI, age, gender, and FOV size as the predictors. A *p*-value less than 0.05 was considered statistically significant.

## 3. Results

### 3.1. Study Population

**Figure 1:**
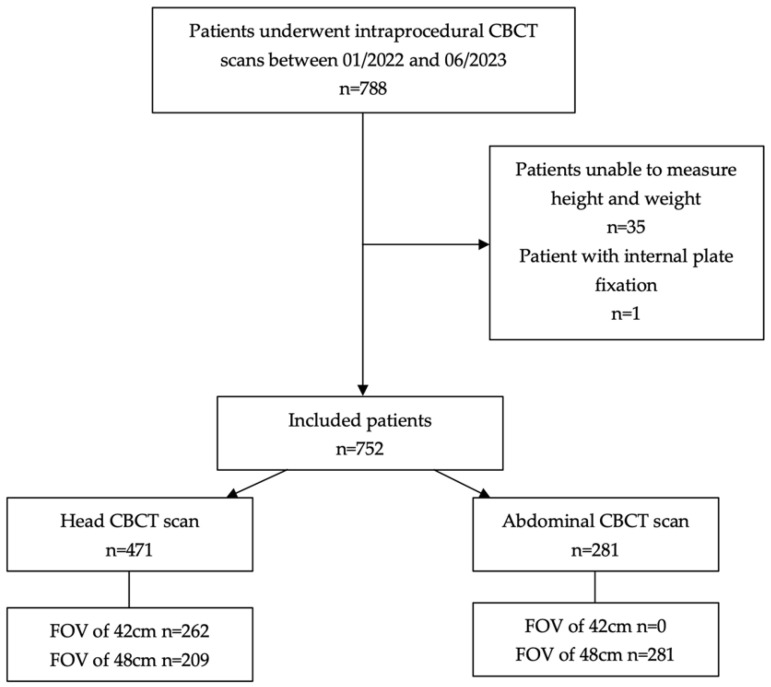
Flowchart of the study. CBCT = cone-beam computed tomography; FOV = field of view.

During the study period, a total of 788 patients underwent intraprocedural CBCT scans. Thirty-five patients were excluded due to unconsciousness and an inability to measure height and weight. One patient was excluded because of a lumbar spine fracture with internal plate fixation (Figure 1).

Finally, a total of 752 patients were included in the study, comprising 461 males (61.3%) and 291 females (38.7%). Among these, 471 (62.6%) underwent a CBCT scan of the head (262 of whom were scanned using an FOV of 42 cm, and 209 were scanned using an FOV of 48 cm), and 281 (37.4%) underwent a CBCT scan of the abdomen (all of whom were scanned using an FOV of 48 cm). The age was 61.9 ± 11.6 years, and the BMI was 23.5 ± 3.5 kg·m^−2^.

### 3.2. Assessment of Patient Radiation Dose and Affecting Factors

**Figure 2:**
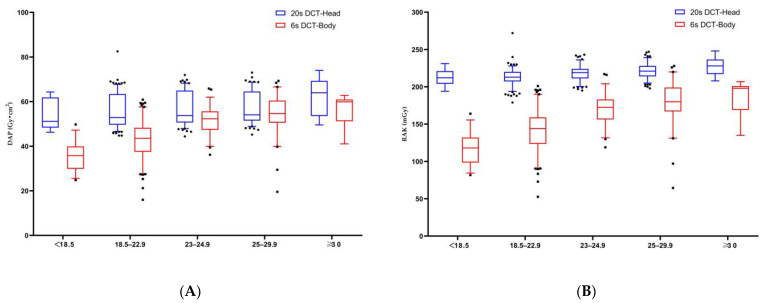
The median values of dose area product (DAP) (**A**) and reference air kerma (RAK) (**B**) showed a stepwise increase across increasing BMI categories in the head and abdominal CBCT scans. The plot shows the interquartile range (box), 5th and 95th percentiles (outermost bars), and median (horizontal bar) of the dose distribution in each group.

Most variables did not have a Gaussian distribution. Therefore, the values are expressed as medians (25th–75th percentile). For a single abdominal CBCT scan, the DAP was 47.4 (39.6–54.3) Gy⋅cm^2^, and the RAK was 156 (130.5–179) mGy. Conversely, for a single head CBCT scan, the DAP was 53.8 (50.5–64.4) Gy⋅cm^2^, and the RAK was 218 (210–224) mGy; these values are 1.1 times and 1.4 times higher, respectively, than those for the abdominal scan. The effects of predictive factors were analyzed and are summarized in Table 2, Table 3, Table 4 and Table 5. Significant increases in both DAP and RAK were observed across increasing BMI categories for all patients (*p*-value < 0.01 for both) (Figure 2). Compared to the patients with a BMI < 18.5, those with a BMI ≥ 30 demonstrated a 1.7-fold increase in DAP for abdominal CBCT (59.9 versus 35.8 Gy⋅cm^2^) and a 1.3-fold increase for head CBCT (63.9 versus 50.8 Gy⋅cm^2^). Additionally, male patients exhibited higher DAP and RAK values in both abdominal and head CBCT scans compared to female patients (*p*-value < 0.001 for both) (Table 2). Notably, when comparing the FOV 48 cm group to the FOV 42 cm group in terms of head CBCT, there was an increase in DAP but a decrease in RAK (*p*-value < 0.001) (Table 3).

### 3.3. Modulation of Exposure Parameters under AEC

**Figure 3:**
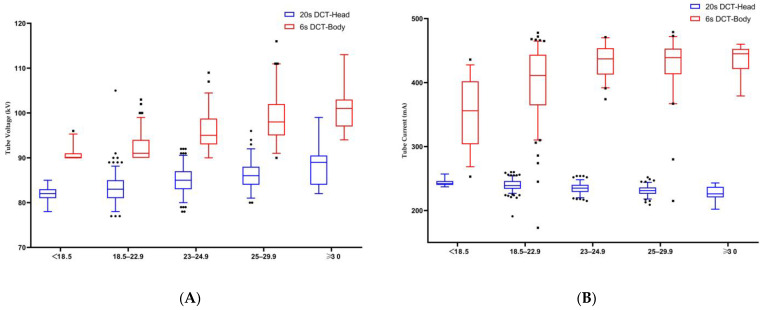
(**A**) Median tube voltage showed a stepwise increase across increasing BMI categories in the head and abdominal CBCT scans; (**B**) median tube current showed a stepwise increase in abdominal CBCT scans and a stepwise decrease in head CBCT scans across increasing BMI categories. The plot shows the interquartile range (box), 5th and 95th percentiles (outermost bars), and median (horizontal bar) of the dose distribution in each group.

The tube voltage and current under AEC adjustment are shown in Table 2, Table 3, Table 4 and Table 5. The male patients had higher tube voltage (*p*-value < 0.001) and tube current (*p*-value < 0.001) values than female patients for abdominal CBCT (Table 2), and both parameters increased with an increasing BMI (with a *p*-value < 0.001 for both) (Figure 3). However, male patients had higher tube voltages (*p*-value < 0.001) and lower tube currents (*p*-value < 0.001) for head CBCT (Table 2), while tube voltages increased (*p*-value < 0.001) and tube currents decreased (*p*-value < 0.001) with an increasing BMI (Figure 3).

### 3.4. Multiple Regression Outcomes

Table 6 shows the results of multiple linear regression analyses conducted for DAP in head and abdominal CBCT, respectively. R^2^ refers to the coefficient of determination and was not adjusted for degrees of freedom. The regression models for DAP were efficient for abdominal CBCT (R^2^ = 0.529) and head CBCT (R^2^ = 0.895). Correlated variables were estimated using standardized coefficients (Std. coef.) and regression coefficients (Reg. coef.). In descending order, patients’ BMI and gender were the independent factors affecting DAP for abdominal CBCT, whereas FOV size, gender, BMI, and age were the independent factors affecting DAP for head CBCT.

## 4. Discussion

The primary objective of this study was to evaluate the clinical radiation dose in patients undergoing CBCT scans of the head and abdomen during interventional procedures and to analyze the independent factors affecting the CBCT radiation dose. The secondary objective was to investigate the influence of relevant factors on CBCT radiation parameters. Since ED cannot be obtained directly during interventional procedures and the results estimated using different methods may vary, the DAP and RAK recommended by the International Commission on Radiological Protection (ICRP) [24] were used as radiation doses in this study.

Our findings revealed that the DAP and RAK for a single rotation of an abdominal CBCT scan were 47.4 (39.6–54.3) Gy⋅cm^2^ and 156 (130.5–179) mGy, respectively. These results are consistent with those from previous studies. Piron et al. [25] investigated radiation exposure via abdominal CBCT during transarterial chemoembolization (TACE) and reported a DAP of 59.397 (46.290–66.401) Gy⋅cm^2^ for a single rotation. Similarly, Berczeli et al. [3] conducted CBCT imaging for visceral aneurysms and found DAP and skin dose values of 57.03 ± 39.67 Gy⋅cm^2^ and 223.6 ± 141.3 mGy, respectively.

At our institution, the protocol used for head CBCT acquisition is 20sDCT-Head, also known as high-resolution CBCT (HR-CBCT). This protocol has proved to be valuable for evaluating intracranial stent expansion in cases of stent-assisted coil embolization as well as for the timely detection of intracranial hemorrhage and early ischemic lesions during procedures [2,26,27]. Compared to 6sDCT-Body, the 20sDCT-Head scans had a higher radiation dose due to having a longer acquisition time (20 s vs. 6 s), more projection images (496 f vs. 397 f), and a higher detector dose (1.200 μGy/fr vs. 0.360 μGy/fr). Our results showed dose values for a single head CBCT scan similar to those in the previous study [2], with 53.8 (50.5–64.4) Gy⋅cm^2^ for DAP and 218 (210–224) mGy for RAK.

Univariate and multivariate analyses were used to analyze the various factors contributing to changes in intraprocedural radiation dose. The results of multiple linear regression showed that patient BMI and gender were independent factors affecting the DAP values in abdominal CBCT scans, while FOV size, gender, BMI, and age were independent factors in head CBCT scans. There was a significant positive correlation between increased patient BMI and higher radiation dose (DAP and RAK) in both abdominal and head CBCT scans. These findings agree with those reported by Suzuki et al. [28], who noted an increase in DAP values as the BMI of phantoms increased during abdominal CBCT. A similar observation was made by Madder et al. [21] during coronary angiography, showing that the radiation dose administered to the patient and physician increased with the patient’s BMI. In addition, BMI was confirmed to be an independent predictor of a peak skin dose (PSD) of over 2 Gy among patients treated for internal carotid aneurysms with pipeline embolization devices [29]. These findings suggest that patient BMI significantly affects the radiation dose in 3D imaging, which is consistent with that of 2D imaging, as dose adjustment automatically keeps the detector signal constant.

However, it should be noted that the influence of BMI on radiation dose varies across different body regions, with there being a more pronounced effect on changes in abdominal CBCT than for head CBCT. Multivariate analysis showed that for each unit increase in BMI, the DAP of the abdominal CBCT scan increased by 1.752 Gy⋅cm^2^_,_ which was about six times higher than the increase in head dose (0.304 Gy⋅cm^2^ per BMI unit). This may be due to the uneven distribution of fat throughout the body. As BMI increases, obese patients, especially those with central obesity, are more likely to have fat concentrated in the abdomen, resulting in a significant increase in body thickness and, therefore, greater dose variability. This observation suggests the need for dose-efficient imaging protocols based on a patient’s BMI, especially for abdominal CBCT scans.

Gender was also identified as a factor affecting the radiation dose administered to patients. Our study showed that male patients received significantly higher radiation doses than female patients in abdominal and head CBCT scans. Previous studies have attributed this difference to higher body weight, more complex diseases, and greater technical difficulties that often be seen in male patients [19,30]. However, the CBCT dose analyzed in our study was generated by a single rotation and therefore independent of procedural time or the complexity of the disease. Klein et al. [20] reported lower radiation doses in female patients undergoing fenestrated endovascular aneurysm repair (FEVAR), and this difference was not associated with patient BMI, operative time, or case complexity, a result that is consistent with our findings. While the exact reasons for this gender discrepancy remain unclear, our study suggests that male patients had greater body weight and height than female patients with similar BMIs, which may lead to increased body thickness and, thus, a greater need for dose compensation.

Furthermore, our study highlights the impact of FOV size on radiation dose. A larger FOV size was associated with significantly higher DAP values, which was expected, as DAP is a product of air kerma and irradiation field area. In the head CBCT scans, the DAP value in the FOV48 cm group was 1.28 times higher than that in the FOV42 cm group. Similar findings have been reported by Kawauchi et al. [16], who found that the DAP value in their FOV27 cm group was 1.44 times higher than that in their FOV22 cm group. However, it is worth noting that their study showed equal RAK values between the two FOV size groups, differing from our results. In our study, the RAK values were higher in the FOV42 cm group than in the FOV48 cm group. This discrepancy may be attributed to differences in the sizes of the patients’ heads, as the cited authors used a phantom for dose evaluation. Additionally, when FOV42 cm was selected, the image was magnified and the input field size was reduced, resulting in decreased brightness. The AEC system then responded by increasing the X-ray dose to maintain constant image brightness. This modulation could explain the increased RAK in the FOV42 cm group. While a large FOV acquisition can visualize an entire object and generate detailed images, a smaller FOV with a smaller voxel size is essential for the accurate and detailed examination of small branches. Therefore, the size of the FOV should be chosen based on the weighing of radiation dose against diagnostic benefits. The use of FOV42 cm reduces the DAP value of a head CBCT scan compared with that for FOV48 cm. Although the RAK value increases with a smaller FOV, the dose dispersion during CBCT rotation limits the deterministic effect on the skin.

Analysis of the exposure parameters with AEC adjustments revealed significant influences of patient BMI and gender on the tube voltage and tube current during CBCT scans. Interestingly, these effects showed different trends during abdominal and head CBCT scans. Although the tube voltage and tube current in scan protocols are set according to application purposes, the AEC dynamically modulates the exposure parameters with reference to the detector entrance dose (DED) [22]. In our institution, the AEC modulation system was set and calibrated by Siemens Healthineers Limited (Shanghai, China) regularly. This attenuation-based modulation process is expected to exhibit significant variability depending on different body regions, patient cross-sections, and the type and settings of the AEC used [22,31]. Our study showed increased tube voltage and tube current for abdominal CBCT and increased tube voltage but decreased tube current for head CBCT for male patients and patients with an increased BMI. This observed variation may be attributed to the modulation priority setting of the AEC controls. Compared to the tube voltage values set in our protocol, the mean kV value for abdominal CBCT scans increased by 3.3% (93 kV compared to 90 kV), while the mean kV value for head CBCT scans increased by 21.4% (85 kV compared to 70 kV). This phenomenon is in accordance with the modulation principles, in that if the initially adjusted tube current fails to reach the desired DED, the tube voltage is adjusted. When the tube voltage increases to a certain level, the tube current decreases. Yel et al. [32] investigated the radiation dose and image quality of an extremity CBCT system with 55 imaging protocols and identified an optimized parameter setting that resulted in a dose reduction of 18.9% compared to the manufacturer’s recommended protocol. Kirisattayakul et al. [33] demonstrated that the implementation of a high kV technique may provide an effective reduction in radiation dose. Our findings show the effects of BMI and gender on exposure parameter modulation in head and abdominal CBCT scans. While most of the parameter settings in the protocol cannot be changed by the user, we can communicate with the manufacturer’s experts to provide additional protocol options based on clinical measurements to optimize patient radiation safety.

There are some limitations of this study. It was a single-center retrospective study with radiation dose data primarily collected from patients undergoing interventional procedures for liver cancer and intracranial aneurysms, which may introduce bias due to the specific population sampled. This bias may be a contributing factor to the non-normal distribution of the dataset. Additionally, there may be interactions between variables, such as weight and gender, that were not fully explored in this study. Thus, more advanced statistics such as mixed effects models should be considered in further research to investigate the interactions between variables. This study demonstrated that FOV size has a significant effect on DAP values in head CBCT. Still, for a complete display of the target, only FOV48 cm is currently used in abdominal CBCT at our institution. To optimize the radiation dose and reduce the risk of stochastic effects, further studies should explore the use of smaller FOV sizes in abdominal CBCT for small-sized patients, especially pediatric patients. Furthermore, only two imaging protocols of one angiograph system were used in this study. Other manufacturers offer different types of AEC systems and settings that may have different modulations of the radiation dose. Research using angiography systems and AEC systems from other manufacturers remains to be performed. Moreover, we evaluated radiation dose and parameters but not image quality in this study. To minimize the radiation dose administered to patients, further studies should explore more optimized parameter protocols for CBCT while maintaining image quality and clinical utility.

## 5. Conclusions

In this study, we evaluated the radiation exposure and influencing factors for head and abdominal CBCT scans used in interventional procedures. This research showed that the analyzed patients received slightly higher radiation doses from head CBCT scans than from abdomen scans during interventional procedures. Patient BMI, gender, and FOV size were the key factors that influenced the radiation dose administered to the patients during CBCT scans. These results may help to define and minimize patients’ exposure to radiation.

## Figures and Tables

**Table 1:** Imaging parameters set for Artis Zeego head and abdominal imaging protocols.

Parameters	20sDCT-Head	6sDCT-Body
Exposure	70 kVp	90 kVp
Pulse width	12.5 ms	5.0 ms
Frame rate	0.4°/F	0.5°/F
Rotation	200°	200°
Imaging start position	98 LAO; 0 CRA	168 RAO; 0 CRA
Number of frames	496 frames	397 frames
Exposure time	20.0 s	6.0 s
Detector size	30 × 40 cm	30 × 40 cm
Field of view	42 cm, 48 cm	48 cm

DCT = Dyna CT; LAO = left anterior oblique; RAO = right anterior oblique; CRA = cranial.

**Table 2:** Radiation exposure data according to gender obtained from head and abdominal CBCT scans.

Protocols	Parameters	Female	Male	*p*-Value
	Number	*n* = 198	*n* = 273	
	DAP (Gy⋅cm^2^)	53.1 (49.6–63.1)	54.2 (51.2–65.6)	<0.0001
20sDCT-Head	RAK (mGy)	212 (206–219)	221 (214–227)	<0.0001
	Tube Voltage (kV)	83 (81–85)	86 (84–88)	<0.0001
	Tube Current (mA)	240 (235–245)	232 (227–237)	<0.0001
	Number	*n* = 93	*n* = 188	
	DAP (Gy⋅cm^2^)	41.0 (34.6–49.0)	50.1 (43.6–55.7)	<0.0001
6sDCT-Body	RAK (mGy)	135 (114.5–161.5)	165 (144–184)	<0.0001
	Tube Voltage (kV)	92 (90–94.5)	95 (91–98.8)	<0.0001
	Tube Current (mA)	398 (346.5–435.5)	432 (398.3–449.5)	<0.0001

Values shown are medians (25th–75th percentile). Analysis was performed using the Mann–Whitney *U*-test. DAP = dose area product; RAK = reference air kerma.

**Table 3:** Radiation exposure data according to FOV size from head CBCT scans.

Protocols	Parameters	FOV 42 cm	FOV 48 cm	*p*-Value
	Number	*n* = 262	*n* = 209	
	DAP (Gy⋅cm^2^)	50.8 (49.1–52.4)	64.8 (62.8–66.9)	<0.0001
20sDCT-Head	RAK (mGy)	220 (213–227)	214 (207–220)	<0.0001
	Tube Voltage (kV)	85 (83–87)	85 (82–87)	0.671
	Tube Current (mA)	235 (230–241)	235 (230–242)	0.874

Values shown are medians (25th–75th percentile). Analysis was conducted using the Mann–Whitney *U*-test. FOV = field of view.

**Table 4:** Radiation exposure data according to BMI category from head CBCT scans.

Parameters	BMI, <18.5	BMI, 18.5–22.9	BMI, 23–24.9	BMI, 25–29.9	BMI, ≥30	*p*-Value
Number	*n* = 10	*n* = 176	*n* = 129	*n* = 139	*n* = 17	
DAP (Gy⋅cm^2^)	50.8 (48.2–62.4)	52.8 (49.6–63.4)	53.7 (50.7–64.9)	54.1 (51.5–64.5)	63.9 (53.6–69.3)	0.001
RAK (mGy)	211.5 (203–220.3)	213 (207.3–220)	219 (211.5–224)	221 (214–228)	228 (217–236.5)	<0.0001
Tube voltage (kV)	82 (80.5–83)	83 (81–85)	85 (83–87)	86 (84–88)	89 (84–90.5)	<0.0001
Tube current (mA)	243.5 (240.8–247.8)	239 (234–246)	235 (229–240)	231 (226–236)	226 (220.5–237)	<0.0001

Values shown are medians (25th–75th percentile). Analysis was conducted using the Kruskal–Wallis *H*-test. BMI = body mass index.

**Table 5:** Radiation exposure data according to BMI category from abdominal CBCT scans.

Parameters	BMI, <18.5	BMI, 18.5–22.9	BMI, 23–24.9	BMI, 25–29.9	BMI, ≥30	*p*-Value
Number	*n* = 33	*n* = 121	*n* = 56	*n* = 60	*n* = 11	
DAP (Gy⋅cm^2^)	35.8 (29.9–39.9)	43.5 (37.5–48.2)	52.3 (47.4–55.6)	54.7 (50.5–60.4)	59.9 (51.2–60.9)	<0.0001
RAK (mGy)	118 (98.5–132)	144 (123.5–159)	172.5 (156.3–183)	180(167–199)	198 (169–201)	<0.0001
Tube voltage (kV)	90 (90–91)	91 (90–94)	95 (93–98.8)	98 (95.3–102)	101 (97–103)	<0.0001
Tube current (mA)	356 (303.5–402)	411 (363–443.5)	437 (412.5–453.8)	439 (413–452.8)	444 (421–453)	<0.0001

Values shown are medians (25th–75th percentile). Analysis was conducted using the Kruskal–Wallis *H*-test.

**Table 6:** Independent factors affecting radiation exposure according to protocol: multiple linear regression results regarding DAP from head and abdominal CBCT scans.

Protocols	Variables	Reg Coef.	Std Coef.	(95% CI)	*p*-Value
6sDCT-Body	Age	−0.001	−0.001	(−0.073/0.071)	0.985
R^2^ = 0.529	Male sex	7.525	0.35	(5.767/9.283)	<0.0001
	BMI	1.752	0.628	(1.525/1.979)	<0.0001
20sDCT-Head	Age	−0.043	−0.066	(−0.062/−0.023)	<0.0001
R^2^ = 0.895	Male sex	2.297	0.151	(1.84/2.753)	<0.0001
	BMI	0.304	0.134	(0.237/0.371)	<0.0001
	FOV 48 cm	13.997	0.926	(13.551/14.443)	<0.0001

“Male sex” is “sex”, and it is shown with respect to female; “FOV 48 cm” is “FOV”, and it is shown with respect to FOV 42 cm. Reg coef. = regression coefficients. Std coef. = standardized coefficients; 95% CI refers to the range of regression coefficients.

## Data Availability

The data are available upon reasonable request from the corresponding author.
